# Practical Facts in Chemistry; Exemplifying the Rudiments, and Showing with What Facility the Principles of the Science May Be Experimentally Demonstrated, at a Trifling Expense, by Means of Simple Apparatus and Portable Laboratories, More Particularly in Reference to Those by R. Best Ede

**Published:** 1837-10

**Authors:** 


Art. X.—*Practical Facts in Chemistry; exemplifying the Rudiments, and showing with what Facility the Principles of the Science may be experimentally demonstrated, at a trifling Expense, by means of simple Apparatus and portable Laboratories, more particularly in reference to those by* Robert Best Ede.—*London*, 1837. 12mo. pp. 193.

It can hardly be expected, nor is it perhaps to be desired, that medical students should enter very deeply into the vast and attractive science of chemistry. A general and experimental knowledge of it, however, they ought to possess; but they are often intimidated from endeavouring to obtain even this limited insight of chemistry, from the great expense of the required apparatus, and the too costly experiments which are described by most writers. In this little work the apparatus described, and the experiments recommended to the student, are cheap and simple; the technical phraseology, too, is reduced to the most familiar and simple form consistent with the dignity of chemistry. For these reasons we can recommend the work to students, as an instructive and at the same time amusing guide.

